# Soluble CD46 improves detection of hepatic steatosis by contrast-enhanced computed tomography before liver surgery

**DOI:** 10.1097/JS9.0000000000002422

**Published:** 2025-04-25

**Authors:** Paul Kupke, Laura S. Kupke, Gunther Glehr, Katja Evert, Ingo Einspieler, Matthias Hornung, Christian Stroszczynski, Edward K. Geissler, Hans J. Schlitt, James A. Hutchinson, Jens M. Werner

**Affiliations:** a Department of Surgery, University Hospital Regensburg, Regensburg, Germany; b Department of Radiology, University Hospital Regensburg, Regensburg, Germany; c Institute of Pathology, University of Regensburg, Regensburg, Germany

**Keywords:** contrast-enhanced computed tomography, preoperative imaging, soluble CD46, steatotic liver disease, surgery complications

## Abstract

Soluble CD46 (sCD46) has recently been shown to be an effective biomarker of steatotic liver disease (SLD), which is highly prevalent in the western world. Since SLD directly affects outcomes after major hepatectomies and liver transplantation, accurate appraisal of this condition is crucial before liver surgery. In the present study, we asked whether sCD46 measurement in patient plasma could augment the pre-surgical prediction of SLD performed by contrast-enhanced computed tomography. Our data show that combined performance of these tests reveals an area under the curve for the prediction of high-grade SLD of 0.956. After cross-validation, our clinical score leads to an accuracy of 95.12% to detect severe steatosis. Therefore, sCD46 is a promising biomarker for optimizing pre-surgical decision-making assessments, with the aim to identify patients that could benefit from alternative treatment options.

## Introduction

Steatotic liver disease (SLD) represents an increasing burden for health systems globally, as it nowadays affects more than a third of the population in most regions, and its prevalence is increasing^[^1^]^. The disease usually progresses silently, predisposing to severe chronic liver damage^[^1^]^. The SLD epidemic also has important implications for liver surgery because the extent of resection is limited by the quality of the remaining organ^[^2^]^. In patients with severe steatosis, appropriate oncological safety margins sometimes cannot be maintained, leading to higher recurrence rates or even inoperability to avoid post-hepatectomy liver failure^[^2,3^]^.

**HIGHLIGHTS**

- We show that presurgery contrast-enhanced computed tomography (CECT) combined with sCD46 titers from blood discriminate high-risk steatosis with an area under the curve of 0.956.
- We provide a cross-validated clinical score incorporating CECT and sCD46 titers which predicts high-risk steatosis with an accuracy of 95.12%.

Preoperative staging of patients with a liver mass is usually performed with a contrast enhanced CT scan (CECT) in order to know the patient’s individual liver anatomy, especially regarding vascular anatomy and dimensions of the tumor^[^4^]^. Unfortunately, detection and quantification of liver steatosis using CECT are less precise and thresholds for inoperability are not defined^[^2,5^]^.

We recently identified soluble CD46 (sCD46) as an accurate biomarker to predict hepatic steatosis, which outperforms established scores like Fatty Liver Index^[^6,7^]^. Hence, the aim of this study was to assess whether sCD46 could improve the performance of preoperative CECT in detecting and quantifying SLD, so as to better identify patients at risk of developing perioperative and postoperative complications during major hepatectomy.

## Methods

This study was performed as a follow-up study to the initial description of sCD46 as a biomarker for hepatic steatosis which included patients who underwent liver resection at the University Hospital Regensburg between August 2014 and September 2019 (Supplemental Digital Content Table 1, available at: http://links.lww.com/JS9/E87). The study was authorized by the Ethics Committee of the University of Regensburg (votum 13-257-101) and registered at clinicaltrials.gov (NCT04943978). This single-center study was performed in accordance with the Declaration of Helsinki and all other applicable laws and ethical standards. All patients provided full, informed verbal and written consent to participate. Plasma samples were collected and stored at −80°C until further use. sCD46 levels were measured as recently described^[^6^]^.

To assess hepatic steatosis in preoperative CECT, five regions of interest (ROI) were defined for each liver and spleen in both arterial and portal venous contrast phases using the clinic’s picture archiving and communication system (Sectra, Sweden). Each ROI was set in different layers of the organ whilst avoiding large vessels and liver parenchyma affected by the primary disease, such as tumor-related cholestasis (Supplemental Digital Content Figure 1, available at: http://links.lww.com/JS9/E87). The median Hounsfield unit (HU) value was then calculated from all five ROI. Histopathological assessment of hepatic steatosis was conducted as recently described^[^6^]^ prior to further analyses. Liver parenchyma with <5% steatosis was considered to be non-steatotic. Steatosis was categorized in grades 1–3 following the cut-offs of 5%, 33%, and 66%, respectively. The distribution within the study population is shown in Supplemental Digital Content Table 1, available at: http://links.lww.com/JS9/E87.

Detailed methods are provided as Supplemental Digital Content, available at: http://links.lww.com/JS9/E88.

## Results

### CECT is useful in predicting liver steatosis of any grade

**Figure 1.**
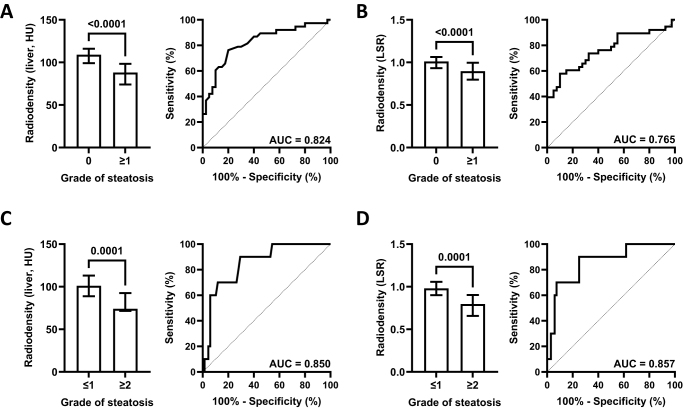
Quantification of hepatic steatosis by contrast-enhanced computed tomography in portal venous phase by assessment of (A) liver and (B) liver-to-spleen ratio (LSR) for any grade of steatosis, (C) liver and (D) LSR for high grades of steatosis (grade 0, *n* = 41; grade 1, *n* = 30; grade ≥2, *n* = 11). Median with interquartile range and receiver operating characteristic curve with area under the curve (AUC) are shown. Groups were compared using Mann–Whitney test.

First, we assessed performance of CECT in predicting any grade of SLD. Patients with histopathological proof of SLD showed lower HU values in the liver parenchyma during portal venous phase than patients without SLD (median 109 vs. 88, *P* < 0.0001) (Fig. 1A). Liver radiodensity performed very well as a discriminator of steatosis (area under the curve [AUC] = 0.824). The optimal cut-off value for diagnosing steatosis was calculated using Youden’s index as 98.5 HU, giving a test sensitivity of 76.32% and specificity of 80.00%. Patients with SLD of any grade also had a lower liver-to-spleen ratio (LSR, median 1.01 vs. 0.90, *P* < 0.0001) (Fig. 1B). LSR performed well as a marker for SLD patients (AUC = 0.765). Setting a cut-off value of 0.905 gave a sensitivity of 57.89% and specificity of 90.00%. CECT during arterial phase showed an inferior performance in the identification of SLD (Supplemental Digital Content Figure 2, available at: http://links.lww.com/JS9/E87, Supplemental Digital Content Table 2, available at: http://links.lww.com/JS9/E87).

### Performance of CECT in predicting severe liver steatosis

Next, we analyzed the performance of CECT to predict high-grade SLD, defined as grade ≥2. By evaluating only the liver tissue, patients with high-grade SLD displayed lower HU values than patients with steatosis grade ≤1 (median 101 vs. 74, *P* = 0.0001) (Fig. 1C). Liver radiodensity discriminated very well between patients with high- or low-grade steatosis (AUC = 0.850). For high-grade SLD, the optimal diagnostic cut-off was 94.5 HU, which gave a test sensitivity of 90.00% and specificity of 70.59%. Likewise, high- and low-grade steatosis could be discriminated by LSR (median 0.98 vs. 0.80, *P* = 0.0001; AUC = 0.857) (Fig. 1D). Setting a cut-off value of 0.905 led to a test sensitivity of 90.00% and specificity of 75.00%.

### Optimal detection of severe SLD by CECT through combination of LSR and sCD46

**Figure 2.**
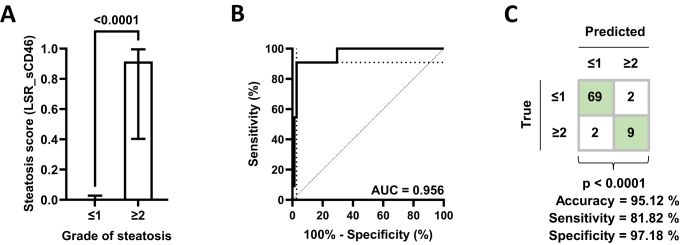
Logistic regression model including liver-to-spleen ratio (LSR) and sCD46 to quantify high-grade SLD with 10-fold cross-validation (grade ≤1, *n* = 71; grade ≥2, *n* = 11). (A) Median with interquartile range. Groups were compared using Mann–Whitney test. (B) Receiver operating characteristic curve with area under the curve (AUC) are shown. An optimal ROC-based cut-off value of 0.345 was set. (C) Cross-tabulation of predicted steatosis grades using the cross-validated score. Groups were compared using Fisher’s exact test.

Next, we further improved SLD quantification by CECT using a logistic regression model incorporating LSR and plasma sCD46 levels (Fig. 2A). Our final model, constructed using the complete dataset, led to a composite score and ROC-based cut-off of 0.345:

$$score=-1.51-0.10\cdot \frac{Liver\left[HU\right]}{Spleen\left[HU\right]}+0.22\cdot sCD46\left[\frac{ng}{ml}\right]$$

$$P\left(severe\;\;steatosis\right)=\frac{1}{1+e^{-score}}$$

The model’s performance was assessed using 10-fold cross-validation (Supplemental Digital Content Table 3, available at: http://links.lww.com/JS9/E87). Our score is an excellent discriminator (cross-validated AUC = 0.956) of high-grade SLD (Fig. 2B). LSR and sCD46 together significantly improve the cross-validated AUC (0.798 vs. 0.956, DeLong *P*-value = 0.047). In cross-validation, we achieved a sensitivity of 81.82%, specificity of 97.18%, and accuracy of 95.12% (Fig. 2C).

## Discussion

CECT is a crucial part in the surgical work-up of oncological patients listed for liver resection. Its role in assessing the general quality of liver parenchyma is often neglected, as native CT phases usually outperform contrast-enhanced phases^[^5^]^. However, to minimize radiation, native CT phases are commonly omitted. As SLD is a major risk factor for perioperative complications^[^2^]^, there is significant advantage in optimizing preoperative detection, both in terms of reducing morbidity and limiting avoidable in-patient costs.

CT remains the cross-sectional imaging modality of choice to detect SLD. In a Japanese study^[^8^]^, a cut-off for native LSR of 1.1 returned a high sensitivity (83.3%) and specificity (81.5%) in detecting ≥30% steatosis. Our study found that CECT leads to a comparable performance, detecting grade ≥2 steatosis with a sensitivity of 90.0% and specificity of 75.0% for the LSR cut-off 0.9.

The role of noninvasive biomarkers in grading SLD is currently the subject of debate^[^6^]^. We recently identified sCD46 as a promising tool to simply predict the grade of steatosis from blood – outperforming established liver parameters and composite scores^[^6^]^. Therefore, we combined both CECT radiodensities and sCD46 levels into a composite score. After cross-validation, our model had an accuracy of 95.1% for predicting high-grade steatosis. Thus, we demonstrated that sCD46 measurements significantly strengthen the informative value of preoperative CECT in diagnosing high-grade SLD. Besides preparation for major liver resection, our score could also be extremely valuable in liver transplantation, where assessment of liver fitness generally falls to the explanting surgeons and is usually one of the critical points for allocation of liver grafts^[^9–11^]^. Hepatic steatosis is a common finding in marginal organs and is strongly associated with elevated risk for primary liver graft non-function^[^9^]^. Using our score, allocation procedures could be optimized using resources already available, without undue delay in donor evaluation.

Our study presents a simple and straightforward method for reliable detection of high-grade steatosis that could improve preoperative assessments, especially in allocating liver transplants and avoiding complications for patients undergoing major liver resection. Follow-up studies are needed to assess the power of sCD46 combined with magnetic resonance imaging (MRI), which currently is the most accurate imaging modality to assess hepatic steatosis^[^12^]^. Since MRI data were not available for our study cohort due to the fact that MRI is not routinely performed pre-surgically in our clinic, this is a limitation of our study.

In the future, our composite score may be useful in identifying patients who might benefit from SLD treatment prior to surgery, or those who should be considered for neoadjuvant chemotherapy instead of upfront surgery.

## Data Availability

Datasets generated during and/or analyzed during the current study are available upon reasonable request.

## Research registration unique identifying number (UIN)

The study was registered at clinicaltrials.gov (NCT04943978).
